# High maternal-fetal HLA eplet compatibility is associated with severe manifestation of preeclampsia

**DOI:** 10.3389/fimmu.2023.1272021

**Published:** 2023-11-03

**Authors:** Katarzyna Stefańska, Małgorzata Kurkowiak, Karolina Piekarska, Elżbieta Chruściel, Dorota Zamkowska, Joanna Jassem-Bobowicz, Przemysław Adamski, Renata Świątkowska-Stodulska, Anna Abacjew-Chmyłko, Katarzyna Leszczyńska, Maciej Zieliński, Krzysztof Preis, Hanna Zielińska, Bogusław Tymoniuk, Piotr Trzonkowski, Natalia Maria Marek-Trzonkowska

**Affiliations:** ^1^ Department of Gynecology and Obstetrics Medical University of Gdansk, Gdańsk, Poland; ^2^ International Centre for Cancer Vaccine Science (ICCVS), University of Gdańsk, Gdańsk, Poland; ^3^ Laboratory of Immunology and Clinical Transplantology, University Clinical Centre in Gdańsk, Gdańsk, Poland; ^4^ Department of Medical Immunology, Medical University of Gdansk, Gdańsk, Poland; ^5^ Laboratory of Immunoregulation and Cellular Therapies, Department of Family Medicine, Medical University of Gdansk, Gdańsk, Poland; ^6^ Department of Neonatology, Medical University of Gdansk, Gdańsk, Poland; ^7^ Department of Endocrinology and Internal Medicine, Medical University of Gdansk, Gdańsk, Poland; ^8^ Department of Immunology and Allergy, Medical University of Łódź, Łódź, Poland

**Keywords:** preeclampsia, gestational hypertension, HLA eplet mismatch load, maternal-fetal HLA compatibility, maternal-fetal immune tolerance, immunology of pregnancy

## Abstract

**Introduction:**

Preeclampsia is responsible for more than 70 000 and 500 000 maternal and fetal deaths, respectively each year. Incomplete remodelling of the spiral arteries in placenta is the most accepted theory of preeclampsia pathogenesis. However, the process is complexed with immunological background, as pregnancy resembles allograft transplantation. Fetus expresses human leukocyte antigens (HLA) inherited from both parents, thus is semiallogeneic to the maternal immune system. Therefore, induction of fetal tolerance is crucial for physiological outcome of pregnancy. Noteworthy, the immunogenicity of discordant HLA antigens is determined by functional epitopes called eplets, which are continuous and discontinuous short sequences of amino acids. This way various HLA molecules may express the same eplet and some HLA incompatibilities can be more immunogenic due to different eplet combination. Therefore, we hypothesized that maternal- fetal HLA incompatibility may be involved in the pathogenesis of gestational hypertension and its progression to preeclampsia. We also aimed to test if particular maternal-fetal eplet mismatches are more prone for induction of anti- fetal HLA antibodies in gestational hypertension and preeclampsia.

**Methods:**

High resolution next-generation sequencing of HLA-A, -B, -C, -DQB1 and -DRB1 antigens was performed in mothers and children from physiological pregnancies (12 pairs) and from pregnancies complicated with gestational hypertension (22 pairs) and preeclampsia (27 pairs). In the next step HLA eplet identification and analysis of HLA eplet incompatibilities was performed with in silico approach HLAMatchmaker algorithm. Simultaneously maternal sera were screened for anti-fetal HLA class I, class II and anti-MICA antibodies with Luminex, and data were analyzed with HLA-Fusion software.

**Results:**

We observed that high HLA-C, -B, and DQB1 maternal-fetal eplet compatibility was associated with severe preeclampsia (PE) manifestation. Both quantity and quality of HLA epletmismatches affected the severity of PE. Mismatches in HLA-B eplets: 65QIA+76ESN, 70IAO, 180E, HLA-C eplets: 193PL3, 267QE, and HLA-DRB1 eplet: 16Y were associated with a mild outcome of preeclampsia if the complication occurred.

**Conclusions:**

High HLA-C, HLA-DQB1 and HLA-B eplet compatibility between mother and child is associated with severe manifestation of preeclampsia. Both quantity and quality of maternal-fetal HLA eplet mismatches affects severity of preeclampsia.

## 1 Introduction

Hypertensive disorders (HDs) are the most common complications of pregnancy (10%) (1). They contribute to increased maternal and fetal morbidity and mortality. HDs can be divided into four categories: chronic hypertension (diagnosed before pregnancy), gestational hypertension (GH), preeclampsia/eclampsia (PE/Ecl), and preeclampsia superimposed on chronic hypertension (2). Among them, GH and PE/Ecl are directly associated with the pregnancy itself, and early child delivery is currently their only cure (3).

GH is diagnosed when elevated systolic (SBP; ≥140 mmHg) or diastolic blood pressure (DBP; ≥90 mmHg) is recorded at least twice after the 20^th^ gestational week and lasts ≥4h (4). In some patients (~25% of GH cases; 3-7% of all pregnancies), GH progresses into PE, which is associated with endothelial dysfunction and changes in the coagulative system that may lead to end-organ injury. The clinical manifestation of PE is usually characterized by proteinuria, elevated levels of liver enzymes, and epigastric pain being the consequence of liver dysfunction, low platelet counts, and/or neurological symptoms (3, 5, 6). Thus, it may contribute to maternal morbidity including renal failure, liver rupture, stroke, eclampsia, and death. In addition, the disorder significantly increases the risk of premature delivery, intrauterine fetal growth restriction (FGR), and perinatal mortality (3, 7, 8).

Despite PE being annually responsible for more than 70,000 maternal deaths and 500,000 fetal deaths globally^5^, its pathogenesis remains unclear. Among several theories, defective placentation and incomplete remodeling of the spiral arteries in the placenta are the most accepted (6, 8–11).

Spiral artery remodeling is a complex process regulated by both fetal and maternal cells. It was observed that extravillous placental trophoblast cells (EVTs) remove the smooth muscle cells from maternal spiral arterioles. Thus, the parts of the vessels located at the maternal-fetal interface are unable to constrict and remain wide open. As a result, a high arteriole capacity and low resistance are reached at the maternal–fetal interface that provides efficient maternal-to-fetal nutrient exchange (8, 12). The process requires the combined engagement of immune cells. Decidual natural killer cells (dNKs) were shown to secrete cytokines and proangiogenic factors crucial for proper vascular remodeling and placentation (11). In addition, recently we and others showed an association between certain combinations of maternal killer cell immunoglobulin-like receptors (KIRs) and fetal/placental HLA-C antigens in the etiology of PE (6, 11).

Pregnancy is a unique physiological state. The fetus shares half of the genome with the mother and half with the father, thus it is semi-allogeneic to the maternal immune system. In this sense, pregnancy resembles allograft transplantation. Therefore, induction of fetal tolerance is crucial for fetal survival and development and full-term pregnancy. It has also been postulated that impaired placentation and PE in general result from insufficient maternal tolerance to fetal alloantigens. This is in accordance with altered proportions and function of NK cell subsets in peripheral blood (3, 6, 13–15) and placentas of women with PE (14, 16). Similar changes were observed for decidual macrophages (17), dendritic cells (DC) (18), and T cells including an increased Th17/Treg ratio (19, 20). In general, PE is recognized as an excessive maternal inflammatory response to the fetus (21). Thus, the previous studies postulated that the pathogenesis of the disease is associated with the recognition of foreign fetal antigens by the maternal immune system. While HLA molecules are the most immunogenic molecules responsible for allogenic graft rejection (22), we hypothesized that fetal HLA antigens are recognized by the maternal immune system thus leading to PE.

Therefore, in the current study, we performed high-resolution next-generation sequencing (NGS) of HLA-A, -B, -C, -DQB1, and -DRB1 antigens of mothers and children from physiological pregnancies and pregnancies complicated with GH and PE. Nevertheless, differences in HLA between donor and recipient (in this case, fetus and mother, respectively) cannot serve for direct prediction of the immune response of the recipient. This results from the way the immune system recognizes self and non-self HLA. The immunogenicity of discordant HLA antigens is determined by functional epitopes called eplets, which are continuous and discontinuous short sequences of amino acids. Various HLA molecules may express the same eplet or the same combinations of eplets. Moreover, particular donor-recipient eplet incompatibilities were shown to be associated with an increased risk of graft rejection, while the others are not immunogenic. Based on this principle, the eplets, but not entire HLA molecules should be analyzed in the context of immune response to non-self-tissues (23–29).

Retrospective studies on oocyte donation pregnancies reported that PE was associated with increased numbers of HLA maternal-fetal mismatches (30). Simultaneously, previous reports on spontaneously conceived pregnancies are contradictory in terms of HLA mismatches and PE onset (31, 32). However, these studies did not analyze maternal-fetal HLA mismatches at the eplet level. Therefore, in the present study, we decided to use an HLAMatchmaker algorithm (26–29) to test if the quantity and quality of maternal-fetal HLA eplet incompatibilities are associated with PE onset.

Unexpectedly, we observed that maternal-fetal incompatibility is a factor protecting from PE, while induction of anti-HLA antibodies in mothers is not associated with the disorder. These results are in line with natural selection theory that favors a diversity of phenotypes, and these findings provide a novel insight into the immunology of pregnancy.

## 2 Materials and methods

### 2.1 Patients

The study comprised 61 pairs of women and their children (122 individuals). The women were divided into healthy controls (n= 12), GH (n=22), and PE (n=27) groups based on clinical and laboratory evaluation according to the International Society for the Study of Hypertension in Pregnancy (ISSHP) classification (33). GH was recognized as hypertension arising *de novo* at ≥ 20 weeks of gestation in the absence of proteinuria or other premises suggestive of PE. Hypertension was diagnosed based on an average of at least two measurements where SBP and/or DBP reached ≥ 140 mmHg and ≥ 90 mmHg, respectively. The patients were assigned to the PE group when gestational hypertension was accompanied by one or more of the following new-onset conditions at ≥20 weeks’ gestation: 1. proteinuria (urine protein creatinine ratio- UPCR ≥ 30 mg/mmol), and 2. other maternal end-organ dysfunction, including neurological complications, pulmonary edema, hematological complications (e.g. platelet count <150 000/μL, disseminated intravascular coagulation- DIC, hemolysis), acute kidney injury (e.g. creatinine ≥90 μmol/L or 1 mg/dL), liver involvement (e.g., elevated ALT or AST *>* 40 IU/L) with or without right upper quadrant or epigastric abdominal pain and uteroplacental dysfunction (e.g., placental abruption, angiogenic imbalance, fetal growth restriction- FGR, abnormal umbilical artery Doppler waveform analysis, or intrauterine fetal death) (33). The exclusion criteria for all groups were: fetal anomalies, asymptomatic bacteriuria or pyelonephritis, kidney diseases, congenital anomalies of kidneys and kidney vessels, chronic arterial hypertension, cardiac diseases, diabetes mellitus, autoimmune conditions, collagenosis, endocrine disorders, thromboembolism, asthma, previous pregnancy/abortion, organ transplantation, and blood transfusion.

The clinical characteristics of the groups are shown in **Table 1**. In addition, we used a scoring system for diversification of PE severity (**Table 2**). The parameters selected for the scoring system overlap with those recommended for PE diagnostics by the International Society for the Study of Hypertension in Pregnancy (ISSHP) (33). For each parameter, the defined value or range of the values had the score assigned. The PE severity was determined after summing up scores for all parameters. The score 5 was established as a cutoff value between mild (≤5) and severe (>5) PE cases.

**Table 1:** Clinical characteristics of the studied groups.

Parameter	Control (n=12)	GH (n=22)	PE (n=27)
Age [years]	29 (24-32)	30 (23-36)	28 (21-36)
SBP [mmHg]	115 (100-125)	160 (120-185)¤	167 (120-193)*
DBP [mmHg]	70 (67-80)	106 (80-120)¤	110 (80-131)*
Proteinuria [G/L]	0	0.11 (0.0-0.56)¤	0.82 (0.09-5.73)*§
Urine creatinine [mmol/L]	N/A	8.3 (4.1-23.23)	6.08 (2.87-17.15)§
UPCR [mg/mmol]	N/A	16.66 (8.17-27.4)	111.87 (13.01-1211.41)§
PLT [x10^4^/μL]	22.15 (17-32.3)	21.2 (15.2-36.1)	20.2 (6.6-33.3)
ALT [U/L]	15.5 (11-32)	11.5 (6-32)	16 (6-233)§
AST [U/L]	23 (14-33)	15 (11-26)	21.5 (13-161)§
Gestational age at the delivery [days]	279 (260-287)	277 (260- 292)	262 (190-281)*§
FGR^a^ [No/Late/Early]	12/0/0	22/0/0	14/3/10

Median, minimum, and maximum values in brackets are given for all the values with the exception of fetal growth restriction (FGR). ^a^number of pregnancies with no FGR, late FGR (onset at ≥32 gestational week; g.w.), and early FGR (onset at <32 g.w). The differences between the three groups were calculated with the Kruskal-Wallis test (KW) for non-parametric data and adjustments were made for multiple comparisons. The Mann-Whitney U test (MW) was used to assess the differences in urine creatinine and UPCR values between GH and PE groups. *Statistically significant differences (p<0.05) between the control and PE groups. § Statistically significant difference (p<0.05) between GH and PE groups. ¤ Statistically significant differences (p<0.05) between the control and GH groups. PLT, platelets; UPCR, urine protein creatinine ratio; SBP, systolic blood pressure; DBP, diastolic blood pressure; ALT, alanine aminotransferase; AST, aspartate aminotransferase.

**Table 2:** Scoring system for diversification of PE severity.

Factor used for score assignment	Score count					
	0	1	2	3	4	5
UPCR [mg/mmol]	<90	90-200	201-400	401-600	601-1000	≥1001
FGR	No	Late	Early	N/A	N/A	N/A
Gestational age at delivery	≥37g.w.+1d	37g.w.-32g.w.+1d	≤32 g.w.	N/A	N/A	N/A
PLT [x10^4^/μL]	≥15	14.9-10	<10	N/A	N/A	N/A
ALT [U/L]	≤33	34-43	44-54	55-100	101-200	>200
AST [U/L]	≤33	34-44	44-54	55-100	101-200	>200
SBP [mmHg]	≤139	140-159	160-179	≥180	N/A	N/A
DBP [mmHg]	≤89	90-99	100-109	≥110	N/A	N/A
Right upper abdomen/epigastric pain	No	Yes	N/A	N/A	N/A	N/A
Neurological complications*	No	Yes	N/A	N/A	N/A	N/A

g.w., gestational week; d, day; FGR, fetal growth restriction; Late FGR- FGR onset at ≥32 g.w; Early FGR- FGR onset at <32 g.w; PLT, platelets; UPCR, urine protein creatinine ratio; SBP, systolic blood pressure; DBP, diastolic blood pressure; ALT, alanine aminotransferase; AST, aspartate aminotransferase; *= headache or blurred vision.

The table lists clinical parameters and their values that were used for the assignment of PE severity scores. The parameters selected for the scoring system overlap with those recommended for PE diagnostics by the International Society for the Study of Hypertension in Pregnancy (ISSHP). For each parameter, a defined value or range of values had the score assigned. The PE severity was determined after summing up scores for all parameters listed in the table.

All participants gave informed consent before enrolment. The study was conducted in accordance with the Declaration of Helsinki and under the protocol approved by the Independent Bioethics Commission for Research of the Medical University of Gdańsk (agreement no. NKBBN/454/2014.)

### 2.2 Sample collection

Samples of peripheral blood for HLA typing, anti-HLA antibody measurement, and analysis of clinical parameters were collected from the mothers before the delivery between 27-41 weeks of gestation, which corresponded with the time of GH and PE onset in the studied groups. In addition, in the case of our patients with pregnancies complicated with PE, the week of PE onset was also the week of child delivery. After delivery, five buccal smear samples were collected from each child using SK-1 swab kits with Isoxelix Dri- Capsules (Isohelix, UK) for HLA typing with NGS.

### 2.3 DNA isolation and HLA typing

HLA-A, -B, -C, -DRB1, and -DQB1 were genotyped using an NGS method on the Illumina platform (Illumina, San Diego, CA, USA) as described in a previous study (25). Sequencing-based HLA typing of the HLA genes -A, -B, -C, -DRB1, and -DQB1 was carried out in 96-well format within a semi-automated workflow by using MiaFora Flex5 typing kits (Immucor, Warren, NJ, USA). Long-range PCR amplification of five HLA loci was performed on Genomic DNA. Genomic DNA from peripheral blood (mothers) and buccal swabs (children) was extracted with the chemagic™ DNA CS200 Kit on the Chemagic 360-D system (Wallac Oy, Mustionkatu 6, FI-20750 Turku, Finland). The results of sequencing were analyzed by MiaFora NGS software v. 4.5, IPD-IMGT/HLA database version 3.40. Data were considered sufficient whenever the coverage reached 40 and the number of cReads exceeded 50,000. The sequencing included the most extensive coverage of the HLA genome, especially with respect to five loci.

### 2.4 HLA eplet identification and analysis of HLA eplet mismatch load

The immunogenicity of HLA mismatches between mother and child was determined with an in silico approach HLAMatchmaker algorithm (Version 3.1) that is freely available online at http://www.epitopes.net/downloads.html. In this approach, eplet incompatibilities for each HLA antigen mismatch are identified and counted to determine HLA immunogenicity. Currently, the HLAMatchmaker is the only tool for the identification of HLA eplet mismatches and analysis of the HLA eplet mismatch load (the number of donor-recipient eplet mismatches). Focus on eplet mismatches rather than on incompatibility between the entire HLA antigens is of great importance for the prediction of the immune response towards non-self HLA. Eplets are the motifs on the HLA molecule surface that are directly recognized and bound by allogeneic antibodies and B-cell receptors (BCR). Thus, the eplets determine HLA immunogenicity. The same eplet can be shared by several HLA antigens and some HLA molecules bear more eplets than others. HLA antigens differ in their immunogenicity when mismatched in allogenic settings (including pregnancy) (25, 34). The International HLA Epitope Registry (www.epregistry.com.br) is a source for the eplet repertoire used by the HLAMatchmaker algorithm. Only experimentally antibody-verified eplets were considered in the analysis (23).

### 2.5 Analysis of antibodies against fetal HLA in maternal sera

First, Luminex anti-HLA and anti-MICA IgG screening was performed with LABScreen Mixed Class I & II test according to the manufacturing procedure with a native serum (One Lambda, Los Angeles CA). The positive result was reported for ≥1.5 scores for anti-HLA class I and ≥2.0 for anti-HLA class II antibodies using the baseline formula. All results were assessed using negative serum (One Lambda, Los Angeles CA). For positive screening results, the specificity of the antibodies was assessed with the LABScreen Single Antigen Test (One Lambda, Los Angeles CA). Then, calculations with HLA-Fusion, ver. 4.6.0 (One Lambda, Los Angeles CA) software were performed according to the baseline formula. Anti-HLA specificities were reported if the result was ≥1000 MFI.

### 2.6 Statistical analysis

Data were calculated with GraphPad Prism software, version 9.4 (673). As data were not normally distributed, the Kruskal-Wallis test (KW), Mann-Whitney U test (MW), and Spearman’s rank correlation (SC) for non-parametric data were used. Values of *p* < 0.05 were deemed significant. Spearman’s rank correlation coefficient (R) reflecting the strength of the correlation is also shown for each SC. The *p* and *R* values are presented in the figures. The gplots package from the R software environment was used for heat map generation.

### 2.7 Data availability

All data required for conclusion evaluation are included in the research paper. HLA sequencing raw data are available upon request from the corresponding author (NMT).

## 3 Results

### 3.1 Higher HLA-C, HLA-DQB1, and HLA-B eplet incompatibility between mother and child is associated with lower PE severity

HLA-A, -B, -C, DRB1, and -DQB1 genes were typed by NGS (**Table 3**) and eplet mismatches were identified for each mother-child pair (**Table 4**).

**Table 3:** HLA-A, -B, -C, -DRB1, and -DQB1 alleles of mother-child pairs from the control, GH, and PE groups.

No.	Group	Mother HLA class I alleles	Child HLA class I alleles	Mother HLA class II alleles	Child HLA class II alleles
1	Control	A*25:01, A*29:02, B*07:02, B*08:01, C*07:01, C*07:02	A*24:02, A*29:02, B*08:01, B*15:01, C*03:03, C*07:01	DRB1*03:01, DRB1*15:01 DQB1*02:01, DQB1*06:02,	DRB1*01:01, DRB1*03:01, DQB1*02:01, DQB1*05:01
2	Control	A*02:01, A*24:02, B*13:02, B*27:02, C*02:02, C*06:02	A*02:01 B*15:01 B*27:02 C*02:02, C*03:03	DRB1*07:01 DRB1*15:01 DQB1*02:02 DQB1*06:02	DRB1*04:01 DRB1*15:01 DQB1*03:02 DQB1*06:02
3	Control	A*01:01 A*02:01 B*49:01 B*52:01 C*07:01 C*12:02	A*02:01 A*11:01 B*49:01 B*51:01 C*07:01, C*14:02	DRB1*11:01 DRB1*15:02 DQB1*03:01 DQB1*06:01	DRB1*04:04 DRB1*11:01 DQB1*03:01 DQB1*04:02
4	Control	A*02:01, B*07:02 B*13:02 C*06:02 C*07:02	A*02:01 A*03:01 B*07:02 B*27:05 C*01:02, C*07:02	DRB1*07:01 DRB1*15:01 DQB1*02:02 DQB1*06:02	DRB1*01:03 DQB1*03:01 DQB1*06:02
5	Control	A*11:01 A*31:01 B*07:02 B*18:01 C*07:01 C*07:02	A*01:01 A*11:01 B*07:02 B*13:02 C*06:02, C*07:02	DRB1*15:01 DRB1*16:01 DQB1*05:02 DQB1*06:02	DRB1*07:01 DRB1*16:01 DQB1*02:02 DQB1*05:02
6	Control	A*02:01 B*13:02 B*15:01 C*03:03 C*06:02	A*02:01 A*26:01 B*15:01 B*58:01 C*03:02, C*03:03	DRB1*07:01 DRB1*13:01 DQB1*02:02 DQB1*06:03	DRB1*03:01 DRB1*13:01 DQB1*02:01 DQB1*06:03
7	Control	A*01:01 A*25:01 B*08:01 B*18:01 C*07:01 C*12:03	A*23:01 A*25:01 B*18:01 B*41:01 C*12:03, C*17:01	DRB1*03:01 DRB1*13:01 DQB1*02:01 DQB1*06:03	DRB1*07:01 DRB1*13:01 DQB1*02:02 DQB1*06:03
8	Control	A*01:01 A*11:01 B*35:01 B*57:01 C*04:01 C*06:02	A*11:01 A*25:01 B*18:01 B*35:01 C*04:01, C*12:03	DRB1*01:01 DRB1*13:05 DQB1*03:01 DQB1*05:01	DRB1*01:01 DRB1*13:01 DQB1*05:01 DQB1*06:03
9	Control	A*02:01 A*03:01 B*07:02 B*39:01 C*07:02 C*12:03	A*03:01 A*33:01 B*07:02 B*14:02 C*07:02, C*08:02	DRB1*11:01 DRB1*15:01 DQB1*03:01 DQB1*06:02	DRB1*01:02 DRB1*15:01 DQB1*05:01 DQB1*06:02
10	Control	A*01:01 A*02:01 B*08:01 B*56:01 C*01:02 C*07:01	A*02:01 A*26:01 B*49:01 B*56:01 C*01:02, C*07:01	DRB1*03:01 DRB1*08:01 DQB1*02:01 DQB1*04:02	DRB1*01:01 DRB1*08:01 DQB1*04:02 DQB1*05:04
11	Control	A*02:01 A*26:01 B*38:01 B*55:01 C*03:03 C*12:03	A*24:02 A*26:01 B*35:01 B*55:01 C*03:03, C*04:01	DRB1*11:03 DRB1*13:01 DQB1*03:01 DQB1*06:03	DRB1*11:01 DRB1*11:03 DQB1*03:01
12	Control	A*02:01 A*02:01 B*27:05 B*57:01 C*01:02 C*06:02	A*02:01 A*02:01 B*14:01 B*57:01 C*06:02, C*08:02	DRB1*01:01 DRB1*04:04 DQB1*03:02 DQB1*05:01	DRB1*04:04 DRB1*07:01 DQB1*02:02 DQB1*03:02
13	GH	A*02:01 A*29:02 B*40:01 B*44:03 C*03:04 C*16:01	A*02:01 A*24:02 B*40:01, C*03:04	DRB1*13:02 DRB1*14:54 DQB1*05:03 DQB1*06:04	DRB1*01:03 DRB1*13:02 DQB1*05:01 DQB1*06:04
14	GH	A*01:01 A*02:01 B*07:02 B*13:02 C*06:02 C*07:02	A*02:01 B*13:02 B*38:01, C*06:02, C*12:03	DRB1*04:01 DRB1*08:01 DQB1*03:01 DQB1*04:02	DRB1*07:01 DRB1*08:01 DQB1*03:03 DQB1*04:02
15	GH	A*02:01 A*03:01 B*14:01 B*47:01 C*06:02 C*08:02	A*02:01 A*24:02 B*14:01 B*40:02 C*02:02, C*08:02	DRB1*07:01 DRB1*11:01 DQB1*02:02 DQB1*03:01	DRB1*07:01 DRB1*11:01 DQB1*02:02 DQB1*03:01
16	GH	A*02:01 A*33:01 B*14:02 B*40:02 C*02:02 C*08:02	A*02:01, B*40:02 B*44:27, C*02:02, C*07:04	DRB1*03:01 DRB1*11:01 DQB1*02:01 DQB1*03:01	DRB1*11:01 DRB1*16:01 DQB1*03:01 DQB1*05:02
17	GH	A*01:01 A*24:02 B*08:01 B*38:01 C*07:01 C*12:03	A*01:01 A*11:01 B*08:01 B*15:01 C*03:03, C*07:01	DRB1*01:01 DRB1*04:04 DQB1*03:02 DQB1*05:01	DRB1*01:01 DRB1*11:03 DQB1*03:01 DQB1*05:01
18	GH	A*02:01 A*03:01 B*35:01 B*41:02 C*04:01 C*17:03	A*03:01 A*31:01 B*35:01 B*45:01 C*04:01, C*06:02	DRB1*01:01 DRB1*13:03 DQB1*03:01 DQB1*05:01	DRB1*01:01 DRB1*07:01 DQB1*02:02 DQB1*05:01
19	GH	A*03:01 A*24:02 B*35:01 B*44:05 C*02:02 C*04:01	A*01:01 A*24:02 B*08:01 B*44:05 C*02:02, C*07:01	DRB1*01:01 DRB1*14:54 DQB1*05:01 DQB1*05:03	DRB1*03:01 DRB1*14:54 DQB1*02:01 DQB1*05:03
20	GH	A*23:01 A*26:01 B*44:03, C*04:01	A*01:01 A*26:01 B*08:01 B*44:03 C*04:01, C*07:01	DRB1*07:01 DQB1*02:02	DRB1*03:01 DRB1*07:01 DQB1*02:01 DQB1*02:02
21	GH	A*01:01 A*02:01 B*18:01 B*57:01 C*06:02 C*07:01	A*02:01 A*74:03 B*18:01, C*07:01, C*12:03	DRB1*07:01 DRB1*14:54 DQB1*03:03 DQB1*05:03	DRB1*12:01 DRB1*14:54 DQB1*03:01 DQB1*05:03
22	GH	A*02:01 A*26:01 B*27:05 B*40:01 C*02:02 C*03:04	A*24:02 A*26:01 B*18:01 B*27:05 C*02:02, C*07:01	DRB1*01:01 DRB1*08:01 DQB1*05:01 DQB1*06:02	DRB1*01:01 DRB1*11:01 DQB1*03:01 DQB1*05:01
23	GH	A*02:01 A*26:01 B*07:02 B*44:03 C*04:01 C*07:02	A*02:01 A*26:01 B*07:02 B*08:01 C*07:01, C*07:02	DRB1*07:01 DRB1*15:01 DQB1*02:02 DQB1*06:02	DRB1*15:01 DRB1*15:01 DQB1*06:02
24	GH	A*01:01 A*03:01 B*08:01 B*38:01 C*07:01 C*12:03	A*01:01, B*08:01, C*07:01	DRB1*03:01 DRB1*13:01 DQB1*02:01 DQB1*06:03	DRB1*03:01 DQB1*02:01
25	GH	A*01:01 A*02:01 B*39:01 B*49:01 C*07:01 C*07:02	A*02:01 A*68:02 B*14:02 B*39:01 C*07:02, C*08:02	DRB1*04:04 DRB1*11:02 DQB1*03:02 DQB1*03:19	DRB1*04:04 DRB1*13:03 DQB1*03:01 DQB1*03:02
26	GH	A*02:01 A*29:02 B*27:02 B*44:03 C*02:02 C*16:01	A*02:01 A*25:01 B*27:02 B*44:02 C*02:02, C*05:01	DRB1*13:02 DRB1*16:01 DQB1*05:02 DQB1*06:04	DRB1*13:01 DRB1*16:01 DQB1*05:02 DQB1*06:03
27	GH	A*01:01 A*26:01 B*08:01 B*35:03 C*07:01 C*12:03	A*01:01 A*26:01 B*08:01 B*39:01 C*07:01, C*12:03	DRB1*04:08 DRB1*15:01 DQB1*03:04 DQB1*06:03	DRB1*15:01 DRB1*16:01 DQB1*05:02 DQB1*06:03
28	GH	A*02:01 A*24:02 B*27:05 B*35:01 C*01:02 C*04:01	A*11:01 A*24:02 B*35:01 B*44:27 C*04:01, C*07:04	DRB1*01:01 DRB1*14:54 DQB1*05:01 DQB1*05:03	DRB1*14:54 DRB1*16:02 DQB1*05:02 DQB1*05:03
29	GH	A*01:01 A*02:01 B*08:01 B*44:02 C*05:01 C*07:01	A*01:01 A*02:01 B*08:01 B*42:02 C*07:01, C*17:01	DRB1*03:01 DRB1*15:01 DQB1*02:01 DQB1*06:02	DRB1*03:01 DRB1*04:01 DQB1*02:01 DQB1*03:04
30	GH	A*01:01 A*02:01 B*08:01 B*44:03 C*04:01 C*07:01	A*02:01 A*24:02 B*15:01 B*44:03 C*03:03, C*04:01	DRB1*11:04 DRB1*13:01 DQB1*03:01 DQB1*06:03	DRB1*07:01 DRB1*11:04 DQB1*03:01 DQB1*03:03
31	GH	A*02:01 A*25:01 B*18:01 B*40:01 C*03:04 C*12:03	A*02:01 A*30:01 B*13:02 B*40:01 C*03:04, C*06:02	DRB1*08:01 DRB1*13:03 DQB1*03:01 DQB1*04:02	DRB1*07:01 DRB1*08:01 DQB1*02:02 DQB1*04:02
32	GH	A*02:01 A*24:02 B*39:01 B*40:01 C*03:04 C*12:03	A*01:01 A*02:01 B*08:01 B*40:01 C*03:04, C*07:01	DRB1*01:01 DRB1*16:01 DQB1*05:01 DQB1*05:02	DRB1*01:01 DRB1*03:01 DQB1*02:01 DQB1*05:01
33	GH	A*02:01 A*24:02 B*07:02 B*08:01 C*07:01 C*07:02	A*01:01 A*24:02 B*07:02 B*44:02 C*07:02, C*16:04	DRB1*01:01 DRB1*03:01 DQB1*02:01 DQB1*05:01	DRB1*01:01 DRB1*04:01 DQB1*03:01 DQB1*05:01
34	GH	A*03:01 A*68:02 B*35:01 B*53:01 C*04:01	A*01:01 A*03:01 B*08:01 B*35:01 C*04:01, C*07:01	DRB1*01:01 DRB1*04:03 DQB1*03:02 DQB1*05:01	DRB1*01:01 DRB1*03:01 DQB1*02:01 DQB1*05:01
35	PE	A*03:01 A*32:01 B*27:07 B*35:03 C*04:01 C*15:02	A*01:01 A*03:01 B*35:03 B*52:01 C*04:01, C*12:02	DRB1*01:01 DRB1*11:04 DQB1*03:01 DQB1*05:01	DRB1*01:01 DRB1*15:02 DQB1*05:01 DQB1*06:01
36	PE	A*24:02 B*07:02 B*39:01, C*07:02 C*07:02	A*23:01 A*24:02 B*07:02 B*49:01 C*07:01, C*07:02	DRB1*11:01 DRB1*15:01 DQB1*03:01 DQB1*06:02	DRB1*11:01 DQB1*03:01
37	PE	A*01:01 A*25:01 B*18:01 B*38:01 C*12:03	A*02:01 A*25:01 B*07:02 B*18:01 C*07:02, C*12:03	DRB1*01:01 DRB1*15:01 DQB1*05:01 DQB1*06:03	DRB1*01:01 DRB1*04:05 DQB1*03:02 DQB1*05:01
38	PE	A*01:01 A*02:01 B*08:01, C*07:01	A*01:01 A*31:01 B*08:01 B*27:05 C*05:01, C*07:01	DRB1*03:01 DRB1*11:01 DQB1*02:01 DQB1*03:01	DRB1*01:01 DRB1*11:01 DQB1*03:01 DQB1*05:01
39	PE	A*02:01 A*11:01 B*15:01 B*56:01 C*01:02 C*03:04	A*02:01 A*33:01	DRB1*04:01 DRB1*09:01 DQB1*03:02 DQB1*03:03	DRB1*09:01 DQB1*03:02
40	PE	A*02:01, B*08:01 B*57:01 C*06:02 C*07:01	A*02:01 A*11:01 B*08:01 B*35:01 C*04:01, C*07:01	DRB1*03:01 DQB1*02:01	DRB1*01:01 DRB1*03:01 DQB1*02:01 DQB1*05:01
41	PE	A*01:01 A*02:01 B*27:05 B*37:01 C*02:02 C*06:02	A*01:01, B*13:02 B*37:01, C*06:02	DRB1*01:01 DRB1*13:02 DQB1*05:01 DQB1*06:04	DRB1*07:01 DRB1*13:02 DQB1*02:02 DQB1*06:04
42	PE	A*02:01, B*41:01 B*44:02 C*05:01 C*17:01	A*02:01, B*44:02 B*51:01 C*05:01, C*14:02	DRB1*04:05 DRB1*11:04 DQB1*02:02 DQB1*03:01	DRB1*11:04 DRB1*13:01 DQB1*03:01 DQB1*06:03
43	PE	A*02:01 A*23:01 B*15:01 B*44:03 C*04:01	A*02:01 A*23:01 B*35:01 B*44:03 C*03:03, C*04:01	DRB1*07:01 DRB1*11:01 DQB1*02:02 DQB1*03:01	DRB1*07:01 DRB1*08:01 DQB1*02:02 DQB1*04:02
44	PE	A*02:01 A*25:01 B*18:01 B*35:03 C*04:01 C*12:03	A*03:01 A*74:03 B*35:01 B*44:03, C*04:01	DRB1*04:01 DRB1*15:01 DQB1*03:02 DQB1*06:02	DRB1*11:01 DRB1*15:01 DQB1*03:01 DQB1*06:02
45	PE	A*02:01 A*31:01 B*07:02 B*39:01 C*07:02 C*12:03	A*01:01 A*02:01 B*07:02 B*27:02 C*02:02, C*07:02	DRB1*04:01 DRB1*15:01 DQB1*03:02 DQB1*06:02	DRB1*11:01 DRB1*15:01 DQB1*03:01 DQB1*06:02
46	PE	A*03:01 A*32:01 B*07:02 B*14:01 C*07:02 C*08:02	A*29:01 A*32:01 B*07:05 B*14:01 C*08:02, C*15:05	DRB1*07:01 DRB1*15:01 DQB1*02:02 DQB1*06:03	DRB1*03:01 DRB1*07:01 DQB1*02:01 DQB1*02:02
47	PE	A*25:01 A*26:01 B*57:01, C*06:02	A*24:02 A*25:01 B*07:02 B*57:01 C*06:02, C*07:02	DRB1*07:01 DQB1*03:03	DRB1*07:01 DRB1*16:01 DQB1*03:03 DQB1*05:02
48	PE	A*24:02 A*26:01 B*35:02 B*44:02 C*04:01 C*05:01	A*24:02, B*07:02 B*35:02 C*04:01, C*07:02	DRB1*03:01 DRB1*13:01 DQB1*02:01 DQB1*06:03	DRB1*03:01 DRB1*11:01 DQB1*02:01 DQB1*03:01
49	PE	A*02:01 A*24:02 B*07:02 B*13:02 C*06:02 C*07:02	A*02:01, B*13:02 B*15:01 C*03:04, C*06:02	DRB1*01:01 DRB1*07:01 DQB1*02:02 DQB1*05:01	DRB1*07:01 DRB1*16:01 DQB1*02:02 DQB1*05:02
50	PE	A*01:01 A*02:01 B*44:02 B*44:03 C*04:01 C*05:01	A*01:01 A*02:01 B*40:01 B*44:02 C*04:01, C*05:01	DRB1*07:01 DRB1*11:04 DQB1*02:02 DQB1*03:01	DRB1*07:01 DRB1*13:02 DQB1*02:02 DQB1*06:04
51	PE	A*03:01 A*32:01 B*08:01 B*27:03 C*02:02 C*07:01	A*02:01 A*03:01 B*35:01 B*56:01 C*01:02, C*04:01	DRB1*03:01 DRB1*11:04 DQB1*02:01 DQB1*03:01	DRB1*12:01 DRB1*15:01 DQB1*03:01 DQB1*06:02
52	PE	A*01:01 A*11:01 B*07:02 B*18:01 C*07:01 C*07:02	A*02:01 A*11:01 B*18:01 B*27:05 C*01:02, C*07:01	DRB1*07:01 DRB1*11:43 DQB1*02:02 DQB1*03:01	DRB1*11:43 DRB1*12:01 DQB1*03:01 DQB1*03:01
53	PE	A*02:01 A*03:01 B*27:05 B*39:01 C*01:02 C*07:02	A*01:01 A*03:01 B*08:01 B*39:01 C*07:01, C*07:02	DRB1*14:54 DRB1*15:01 DQB1*05:03 DQB1*06:02	DRB1*03:01 DRB1*14:54 DQB1*02:01 DQB1*05:03
54	PE	A*03:01 A*24:02 B*15:01 B*44:03 C*03:04 C*04:01	A*02:01 A*24:02 B*40:01 B*44:03 C*03:04, C*04:01	DRB1*11:01 DRB1*15:01 DQB1*03:01 DQB1*06:02	DRB1*08:01 DRB1*11:01 DQB1*03:01 DQB1*04:02
55	PE	A*03:01 A*25:01 B*07:02 B*13:02 C*06:02 C*07:02	A*01:01 A*25:01 B*08:01 B*13:02 C*06:02, C*07:01	DRB1*07:01 DRB1*15:01 DQB1*02:02 DQB1*06:02	DRB1*03:01 DRB1*07:01 DQB1*02:01 DQB1*02:02
56	PE	A*01:01 A*02:01 B*07:02 B*35:03 C*07:02 C*12:03	A*01:01 A*03:01 B*07:02 B*50:01 C*06:02, C*07:02	DRB1*13:01 DRB1*14:54 DQB1*05:03 DQB1*06:03	DRB1*07:01 DRB1*13:01 DQB1*02:02 DQB1*06:03
57	PE	A*01:01 A*29:02 B*44:03 B*57:01 C*06:02 C*16:01	A*01:01 A*32:01 B*27:05 B*57:01 C*01:02, C*06:02	DRB1*07:01 DQB1*02:02 DQB1*03:03	DRB1*07:01 DRB1*13:01 DQB1*03:03 DQB1*06:03
58	PE	A*01:01 A*32:01 B*08:01 B*55:01 C*03:03 C*07:01	A*02:01 A*32:01 B*13:02 B*55:01 C*03:03, C*06:02	DRB1*03:01 DRB1*04:01 DQB1*02:01 DQB1*03:02	DRB1*04:01 DRB1*07:01 DQB1*02:02 DQB1*03:02
59	PE	A*03:01 A*24:02 B*18:01 B*35:01 C*04:01 C*12:03	A*02:01 A*03:01 B*27:02 B*35:01 C*02:02, C*04:01	DRB1*01:01 DRB1*15:01 DQB1*05:01 DQB1*06:02	DRB1*01:01 DRB1*16:01 DQB1*05:01 DQB1*05:02
60	PE	A*02:01 A*03:01 B*51:01 B*51:01 C*05:01 C*15:02	A*02:01 A*03:01 B*18:01 B*51:01 C*07:01, C*15:02	DRB1*12:01 DRB1*15:01 DQB1*03:01 DQB1*06:02	DRB1*07:01 DRB1*12:01 DQB1*02:02 DQB1*03:01
61	PE	A*02:01 A*24:02 B*13:02 B*51:01 C*01:02 C*06:02	A*02:01 A*24:02 B*27:05 B*51:01 C*01:02, C*02:02	DRB1*07:01 DRB1*11:01 DQB1*02:02 DQB1*03:01	DRB1*01:01 DRB1*11:01 DQB1*03:01 DQB1*05:01

The table lists HLA class I and class II alleles typed with the NGS method for each mother and child pair from uncomplicated (control; blue shading) and complicated pregnancies with gestational hypertension (GH; yellow shading) and preeclampsia (PE; red shading).

**Table 4:** HLA-A, -B, -C, DRB1, and -DQB1 eplet mismatches in mother-child pairs from the control, GH, and PE groups.

No.	Group	HLA-A eplet mismatches	No. of HLA-A eplet mismatches	HLA-B eplet mismatches	No. of HLA-B eplet mismatches	HLA-C eplet mismatches	No. of HLA-C eplet mismatches	HLA-DRB1 eplet mismatches	No. of HLA-DRB1 eplet mismatches	HLA-DQB1 eplet mismatches	No. of HLA-DQB1 eplet mismatches
1	Control	62EE, 65GK, 80I+90A, 82LR+90A, 82LR+145RA, 127K, 144K, 144KR, 144KR+127K, 144KR+151H, 150AAH, 166DG	12	44RMA, 131S, 163LW, 163LW+65QIT	4	21H, 163LW, 173K, 193PV, 219W	5	13FE, 96EV	2	37YV, 74SR3, 74SV2, 125SQ	4
2	Control	0	0	69TNT+80N, 71TTS, 76ESN, 80N, 163LW, 163LW+65QIT	6	65QKR+76VS, 76VRN, 80N, 163LW, 173K, 219W	6	37YV, 96Y2	2	52PL3, 55PP	2
3	Control	66NV, 151AHA, 163RW	3	0	0	219W	1	96Y2, 98E, 104A	3	55RL3, 74SV2	2
4	Control	62QE+56G, 66NV, 138MI, 138MI+79GT, 144KR, 144KR+151H, 161D	7	71ATD, 82LR+144QR, 82LR+145R, 82LR+145RA	4	219W, 248M	2	13FE, 70DA, 96EV	3	45EV, 52PL3, 55PP	3
5	Control	44KM3, 76ANT, 163RG, 166DG	4	41T, 44RMA, 80TLR, 82LR, 82LR+90A, 82LR+138T, 144QL	7	73AN, 80K, 80K+14R, 193PV	4	25Q3, 57V, 78V2, 98E, 104A, 181M	6	45GE3, 84QL3	2
6	Control	62RR, 66NV, 76ANT, 79GT+90D, 138MI, 138MI+79GT, 145RT, 149TAH, 163R, 163RW	10	44RT, 62GRN, 69AA, 69AA+76E, 71SA, 80I, 80I+90A, 82LR+144QR, 82LR+145R, 82LR+145RA	10	0	0	74R, 77N	2	0	0
7	Control	62EE, 65GK, 80I+90A, 82LR+90A, 82LR+145RA, 127K	6	41T	1	73AN, 80K, 80K+14R, 163EW	4	25Q3, 57V, 78V2, 98E, 104A, 181M	6	0	0
8	Control	62RR, 76ESI, 82LR+138M, 145RT, 149TAH, 253Q	6	131S+163T	1	65QKR+76VS, 76VRN	2	0	0	0	0
9	Control	62RR	1	0	0	138K, 177KT	2	13FE, 96EV	2	74SR3, 74SV2, 77R, 125SQ	4
10	Control	62RR, 66NV, 145RT, 149TAH, 163RW	5	41T, 80I, 80I+69TNT, 80I+90A, 82LR, 82LR+90A, 82LR+138T, 82LR+144QR, 82LR+145R, 82LR+145RA, 163LW+65QIT	11	0	0	13FE, 70QT, 96EV	3	52PQ2, 74SR3	2
11	Control	62EE, 65GK, 82LR+138M, 144KR, 144KR+127K, 144KR+151H, 166DG	7	44RT, 44RT+69TNT, 69TNT+80N, 71TTS, 163LW+65QIT	5	73AN, 80K	2	0	0	0	0
12	Control	0	0	69TNT, 69TNT+80N, 71TTS, 76ESN, 131S+163T	5	138K, 177KT	2	25Q3, 57V, 70D, 78V2, 181M	5	45GE3	1
13	GH	62EE, 65GK, 80I, 80I+90A, 82LR+138M, 144KR, 144KR+127K, 144KR+151H, 166DG	9	0	0	0	0	13FE, 96EV	2	0	0
14	GH	0	0	80I, 80I+69TNT, 80I+90A, 82LR+144QR, 82LR+145R, 82LR+145RA, 131S+163T, 158T	8	0	0	25Q3, 57V, 78V2, 181M	4	0	0
15	GH	62EE, 65GK, 80I, 80I+90A, 82LR+138M, 144KR+127K, 166DG	7	113H	1	21H	1	0	0	0	0
16	GH	0	0	80TLR, 82LR, 82LR+90A, 82LR+138T, 82LR+144QR, 82LR+145R, 82LR+145RA, 156DA, 163LS/G	9	90D, 156DA, 193PL3, 267QE	4	142M3	1	45GV, 52PQ2, 52PR, 74SR3, 74SV2	5
17	GH	66NV, 151AHA, 163RW	3	44RMA, 163LW, 163LW+65QIT	3	21H, 163LW, 173K, 219W	4	11STS, 47F, 56EDR11, 56EEDR11, 57D, 57DE, 57DEDP, 70D, 70DA, 96HK	10	45EV	1
18	GH	56R	1	163LS/G	1	0	0	25Q3, 57V, 78V2, 98E, 104A, 181M	6	45GE3	1
19	GH	44KM3, 76ANT, 79GT+90D, 163R, 163RG	5	66IF+163TEW, 180E	2	76VRN, 193PL3, 253Q, 267QE	4	47F, 74R, 77N	3	45GE3, 84QL3	2
20	GH	44KM3, 62QE+56G, 144K, 144KR, 144KR+151H, 163RG	6	66IF+163TEW, 69TNT+80N, 71TTS, 76ESN, 80N, 113H, 156DA, 180E	8	76VRN, 80N, 193PL3, 267QE	4	11STS, 25R, 47F, 74R, 77N, 96HK	6	0	0
21	GH	66NV	1	0	0	65QKR+76VS	1	16Y, 37L, 47F, 70DA	4	45EV	1
22	GH	62EE, 65GK, 80I, 80I+90A, 82LR+138M, 144KR, 144KR+127K, 144KR+151H, 166DG	9	44RT, 44RT+69TNT, 131S+163T	3	193PL3, 267QE	2	11STS, 47F, 56EDR11, 56EEDR11, 57DE, 57DEDP	6	45EV, 52PL3, 55PP, 84QL3	4
23	GH	0	0	66IF+163TEW, 69TNT+80N, 71TTS, 156DA	4	0	0	0	0	0	0
24	GH	0	0	0	0	0	0	0	0	0	0
25	GH	62RR, 66NV	2	0	0	138K, 177KT, 193PV	3	0	0	0	0
26	GH	62RR, 76ESI, 82LR+138M, 90D, 145RT, 149TAH, 163R, 163RW	8	156DA	1	138K, 177KT	2	0	0	0	0
27	GH	0	0	131S+163T, 158T	2	0	0	70D, 70DA	2	74SR3, 74SV2, 77R	3
28	GH	62QE+56G, 66NV, 79GT+90D, 138MI+79GT, 151AHA, 163R, 163RW	7	41T, 156DA, 163LS/G	3	156DA, 177KT, 193PL3, 267QE	4	70D, 70DA, 142M3	3	0	0
29	GH	0	0	65QIA, 65QIA+76ESN, 69AA, 69AA+65QI, 69AA+76E, 70IAQ	6	73AN, 163EW	2	37YV, 96Y2, 98E, 104A	4	45EV, 52PL3, 55PP	3
30	GH	62EE, 65GK, 80I, 80I+90A, 82LR+138M, 144KR+127K	6	44RMA, 163LW, 163LW+65QIT	3	21H, 65QKR+76VS, 163LW, 173K	4	25Q3, 57V, 78V2, 98E, 104A, 181M	6	0	0
31	GH	56R, 138MI+79GT	2	44RMA, 80TLR, 82LR+90A, 82LR+138T, 144QL	5	73AN, 80K, 80K+14R	3	25Q3, 57V, 78V2, 98E, 104A, 181M	6	45GE3, 77R, 140A2	3
32	GH	44KM3, 62QE+56G, 76ANT, 79GT+90D, 90D, 138MI+79GT, 163R, 163RG	8	66IF+163TEW, 156DA	2	90D, 193PL3, 267QE	3	11STS, 47F, 74R, 77N, 96HK	5	45GE3, 84QL3	2
33	GH	44KM3, 62QE+56G, 76ANT, 79GT+90D, 138MI+79GT, 163R, 163RG	7	41T, 80TLR, 82LR+138T, 82LR+144QR, 131S, 163LS/G	6	0	0	96Y2, 98E, 104A	3	45EV, 52PL3, 55PP	3
34	GH	44KM3, 76ANT, 79GT+90D, 163R, 163RG, 166DG	6	66IF+163TEW, 156DA, 180E	3	76VRN, 193PL3, 267QE	3	11STS, 47F, 74R, 77N, 96HK	5	45GE3	1
35	PE	44KM3, 76ANT, 79GT+90D, 163R, 163RG, 166DG	6	80I+69TNT	1	65QKR+76VS, 76VRN	2	142M3	1	0	0
36	PE	82LR+144QR	1	41T, 80I+69TNT, 82LR+138T, 82LR+144QR, 163LW, 163LW+65QIT	6	0	0	0	0	0	0
37	PE	43Q+62GER, 62GE, 62GK2, 107W, 127K, 144TKH, 145KHA, 150AAH	8	65QIA, 65QIA+76ESN, 69AA, 69AA+65QI, 69AA+76E, 70IAQ, 163EW, 163EW+66I, 163EW+73TE, 180E	10	193PL3, 267QE	2	96Y2, 98E, 104A	3	52PL3, 55PP, 84QL3	3
38	PE	56R, 66NV	2	65QIA, 69AA, 69AA+65QI, 69AA+76E, 71ATD, 80TLR, 82LR, 82LR+90A, 82LR+138T, 82LR+144QR, 82LR+145R, 82LR+145RA, 131S, 163EW, 163EW+66I, 163EW+73TE	16	80K, 80K+14R, 138K, 177KT, 193PV	5	13FE, 70QT, 96EV	3	45GV, 52PQ2, 52PR, 74SR3, 74SV2, 125SQ	6
39	PE	62RR	1	0	0	0	0	0	0	0	0
40	PE	62QE+56G, 66NV, 79GT+90D, 138MI, 138MI+79GT, 144KR, 144KR+151H, 151AHA, 163R, 163RW	10	44RT, 44RT+69TNT, 163LW+65QIT	3	219W	1	13FE, 70QT, 73A, 77T, 96EV	5	37YV, 45GV, 46VY3, 52PQ2, 52PR, 74SR3, 74SV2, 125SQ	8
41	PE	0	0	41T, 44RMA, 113H, 144QL	4	0	0	25Q3, 57V, 78V2, 98E, 104A, 181M	6	45GE3, 84QL3	2
42	PE	0	0	44RT, 44RT+69TNT, 80I, 80I+69TNT, 80I+90A, 163LW, 163LW+65QIT	7	65QKR+76VS, 76VRN, 219W	3	0	0	45GV, 52PQ2, 52PR	3
43	PE	0	0	44RT, 44RT+69TNT	2	21H, 65QKR+76VS, 76VRN, 173K	4	16Y	1	45GV, 52PR, 55RL3, 74SV2	4
44	PE	62QE+56G, 138MI+79GT, 144KR, 144KR+151H, 161D	5	41T, 80TLR, 82LR+90A, 82LR+138T, 82LR+145RA, 163LS/G	6	0	0	11STS, 56EDR11, 56EEDR11, 57DE, 57DEDP, 70D, 70DA, 96HK	8	45EV	1
45	PE	44KM3, 62QE+56G, 76ANT, 79GT+90D, 144KR, 144KR+151H, 163R, 163RG, 166DG	9	80I, 80I+90A, 82LR, 82LR+90A, 82LR+138T, 82LR+144QR, 82LR+145R, 82LR+145RA	8	21H, 80K, 80K+14R	3	11STS, 56EDR11, 56EEDR11, 57DE, 57DEDP, 70D, 70DA, 96HK	8	45EV	1
46	PE	62LQ, 76ANT	2	0	0	21H, 80K, 80K+14R	3	11STS, 74R, 77N, 96HK	4	0	0
47	PE	62EE, 65GK, 127K, 144K, 144KR, 144KR+127K, 144KR+151H, 150AAH, 166DG	9	65QIA, 65QIA+76ESN, 69AA+65QI, 70IAQ, 76ESN, 80N, 163EW, 163EW+66I, 163EW+73TE, 180E	10	65QKR+76VS, 76VRN, 80N, 193PL3, 267QE	5	25R, 70DA, 73A, 142M3	4	37YV, 52PQ2, 52PR, 74SR3, 74SV2, 77R, 140A2	7
48	PE	0	0	65QIA, 65QIA+76ESN, 69AA, 69AA+65QI, 69AA+76E, 70IAQ, 163EW, 163EW+66I, 163EW+73TE, 180E	10	65QKR+76VS, 76VRN, 193PL3, 267QE	4	37YV, 56EDR11, 56EEDR11, 57DE, 57DEDP	5	45EV, 52PL3, 55PP	3
49	PE	0	0	69TNT+80N, 71TTS, 163LW, 163LW+65QIT	4	21H, 163LW, 173K, 219W	4	70DA, 142M3	2	0	0
50	PE	0	0	69TNT+80N, 71TTS, 76ESN, 80N, 113H, 143S+76ESN, 163EW, 163EW+66I, 163EW+73TE, 180E	10	0	0	57D	1	45GV, 52PQ2, 52PR	3
51	PE	43Q+62GER, 62GE, 62GK2, 107W, 127K, 144TKH, 145KHA	7	44RT, 44RT+69TNT, 65QIA+76ESN, 70IAQ, 163LW, 163LW+65QIT	6	65QKR+76VS, 73AN, 219W, 248M	4	16Y, 37L, 57V, 70QT, 142M3	5	45GV, 52PQ2, 52PR	3
52	PE	43Q+62GER, 62GE, 62GK2, 107W, 127K, 144TKH, 145KHA	7	71ATD, 80TLR, 82LR, 82LR+90A, 82LR+138T, 82LR+144QR, 82LR+145R, 82LR+145RA	8	193PV, 219W, 248M	3	16Y, 37L, 96HK	3	0	0
53	PE	44KM3, 76ANT, 79GT+90D, 163R, 163RG, 166DG	6	66IF+163TEW, 156DA, 180E	3	0	0	74R, 77N	2	45GE3, 84QL3	2
54	PE	43Q+62GER, 62GE, 62GK2, 107W, 144TKH, 145KHA, 253Q	7	143S+76ESN, 163EW, 163EW+66I, 163EW+73TE, 180E	5	0	0	16Y	1	55RL3, 74SV2	2
55	PE	44KM3, 76ANT, 79GT+90D, 163RG, 166DG	5	66IF+163TEW, 69TNT+80N, 71TTS, 156DA	4	0	0	11STS, 74R, 77N, 96HK	4	0	0
56	PE	66NV, 161D	2	41T	1	73AN, 80K, 80K+14R	3	25Q3, 57V, 78V2, 98E, 104A, 181M	6	45GE3, 84QL3	2
57	PE	76ESI, 82LR+138M	2	65QIA, 69AA+65QI, 71ATD, 163EW, 163EW+66I, 163EW+73TE	6	219W, 248M	2	11STS, 25R, 47F, 70DA, 73A, 96HK	6	52PQ2, 52PR	2
58	PE	43Q+62GER, 62GE, 145KHA2, 107W, 127K, 144TKH, 145KHA, 150AAH	8	41T, 44RMA, 80TLR, 82LR+138T, 144QL, 163EW, 163EW+66I, 163EW+73TE	8	73AN, 80K, 80K+14R	3	25Q3, 57V, 70D, 78V2, 181M	5	0	0
59	PE	43Q+62GER, 62GE, 62GK2, 107W, 144TKH, 145KHA, 253Q	7	65QIA, 69AA, 69AA+65QI, 69AA+76E, 82LR+138T, 82LR+144QR, 163EW, 163EW+66I, 163EW+73TE	9	21H, 80K+14R, 163EW	3	70D, 70DA	2	0	0
60	PE	0	0	69TNT+80N, 71TTS, 76ESN, 80N, 131S+163T	5	76VRN, 80N, 90D, 193PL3, 267QE	5	25Q3, 78V2, 98E, 104A, 181M	5	45GE3, 77R	2
61	PE	0	0	65QIA, 69AA, 69AA+65QI, 69AA+76E, 71ATD	5	21H	1	13FE, 70QT, 96EV	3	45GV, 52PQ2, 52PR, 74SR3, 74SV2, 125SQ	6

Antibody verified eplet mismatches in HLA-A, - B, - C, -DRB1, and -DQB1 antigens between mother and child are listed for uncomplicated (control; blue shading) and complicated pregnancies with gestational hypertension (GH; yellow shading) and preeclampsia (PE; red shading). In addition, the number of HLA eplet mismatches (HLA eplet mismatch load) for each studied allele pair is given.

**Figure 1:**
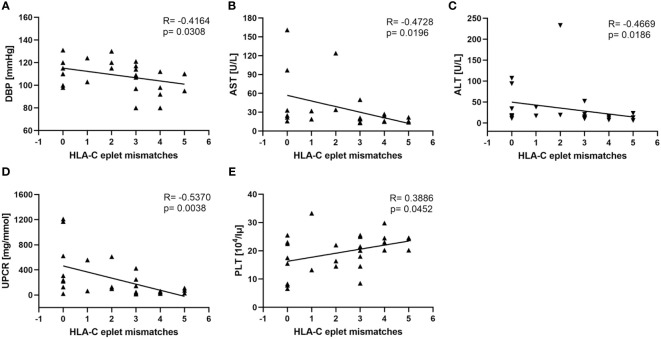
Low maternal-fetal HLA-C eplet incompatibility is associated with worse clinical characteristics of women with PE. Increased DBP **(A)**, AST **(B)**, ALT **(C)**, and UPCR **(D)** values and decreased platelet (PLT) count **(E)** are associated with lower HLA-C eplet mismatch load (n=27) in pregnancies complicated with preeclampsia (PE). Correlations were calculated with Spearman’s rank correlation, and R and p values are given.

**Figure 2:**
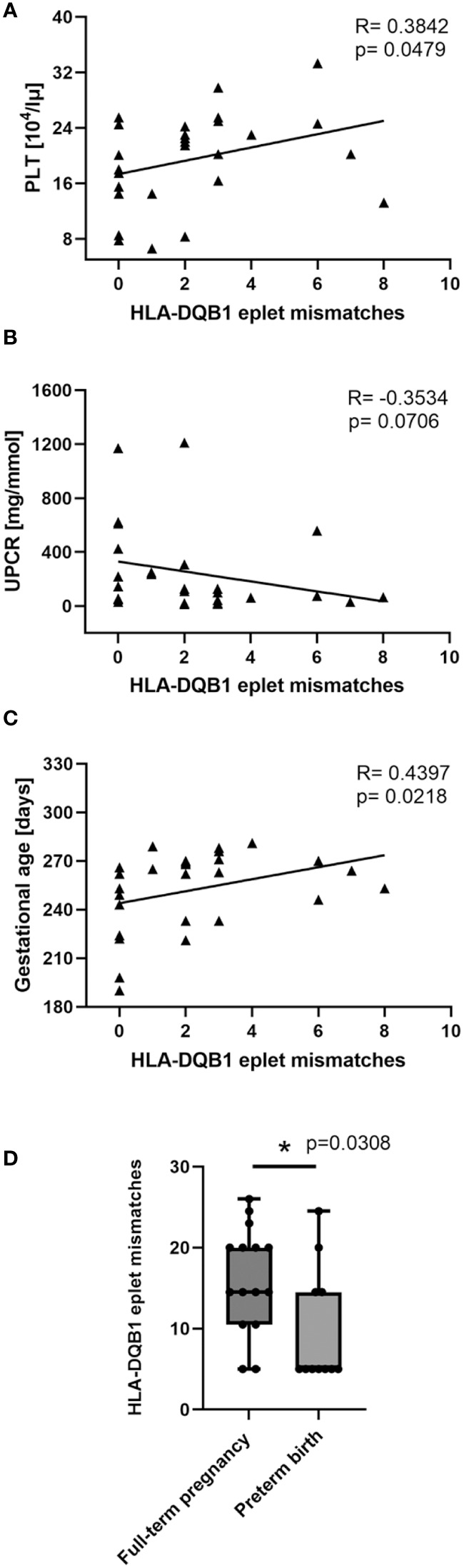
Low maternal-fetal HLA-DQB1 eplet incompatibility is associated with worse clinical characteristics of women with PE. Decreased platelet (PLT) count **(A)**, increased UPCR **(B)** values, and low gestational age at delivery **(C)** are associated with lower HLA-DQB1 eplet mismatch load in pregnancies complicated with preeclampsia (PE). Correlations were calculated with Spearman’s rank correlation, and R and p values are given (n=27, for AST and ALT n=24 and n=25, respectively). In the PE group, full-term deliveries were characterized by significantly higher maternal-fetal HLA-DQB1 eplet incompatibilities than premature deliveries **(D)**. The differences were calculated with the Mann-Whitney U test (n=27, for AST and ALT n=24 and n=25, respectively). The boxplot depicts relative ranks. The medians (symbol within the boxes), quartiles (box), and ranges (whiskers) are shown. *p<0.05 is considered statistically significant.

No statistically significant differences were observed between the groups when numbers of eplet mismatches were compared for HLA-A, -B, -C, -DRB1, and -DQB1. Nevertheless, in PE we observed significant negative correlations for HLA-B, HLA-C, and HLA-DQB1 eplet incompatibilities between mother and child and factors considered to be predictors and hallmarks of PE. The most significant association between PE outcome and eplet mismatches was found for HLA-C antigens. HLA-C eplet mismatch load was in negative correlation with DBP (SC; R= -0.4164, p=0.0308; **Figure 1A**), AST (SC; R= -0.4728, p=0.0196; **Figure 1B**), and ALT (SC; R= -0.4669, p=0.0186 **Figure 1C**). High HLA-C eplet mismatch load between mother and child was also associated with better kidney function as reflected by lower UPCR values (SC; R= -0.5370, p=0.0038; **Figure 1D**). In addition, low HLA-C (**Figure 1E**) and HLA-DQ (**Figure 2A**) eplet incompatibility were associated with low platelet count (PLT), another hallmark of PE (33) (SC; R=0.3886, p=0.0452; **Figure 1E**and R=0.3842, p=0.0479; **Figure 2A**, respectively).

Similar HLA-C, higher eplet mismatch load for HLA-DQB1 antigens was associated with better kidney function (UPCR), but the correlation did not reach statistical significance (SC; R= -0.3534, p=0.0706; **Figure 2B**). In addition, HLA-DQB1 eplet incompatibility correlated positively with gestational age at birth (SC; R=0.4397, p= 0.0218; **Figure 2C**) and was associated with lower frequency of premature deliveries (MW; p=0.0308; **Figure 2D**).

**Figure 3:**
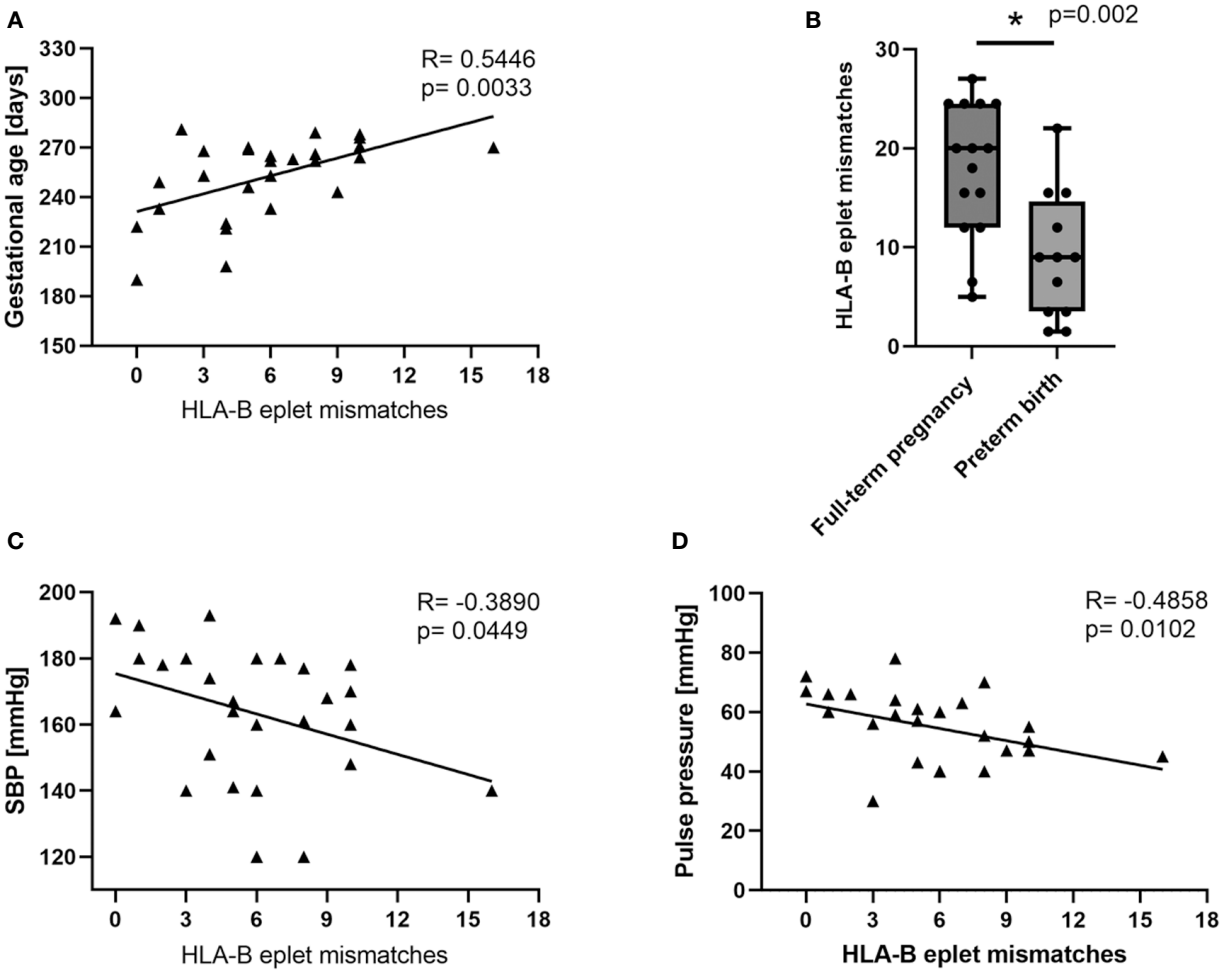
Low maternal-fetal HLA-B eplet incompatibility is associated with worse clinical characteristics of women with PE. Gestational age at delivery correlated positively with HLA-B eplet mismatch load **(A)** in preeclampsia (PE). In the PE group, full-term deliveries were characterized by significantly higher maternal-fetal HLA-B eplet incompatibilities than premature deliveries **(B)**. Maternal-fetal HLA-B eplet mismatch load correlates negatively with systolic blood pressure (SBP; **C**) and pulse pressure **(D)**. Correlations were calculated with Spearman’s rank correlation, and R and p values are given (n=27). Differences between full-term and premature deliveries were calculated with the Mann-Whitney U test (n=27). The boxplot depicts relative ranks. The medians (symbol within the boxes), quartiles (box), and ranges (whiskers) are shown. *p<0.05 is considered statistically significant.

Even stronger correlation between number of eplet mismatches and gestational age was observed for HLA-B antigens (SC; R= 0.5446, p=0.0033; **Figure 3A**). Full-term deliveries were characterized with high number of eplet incompatibilities for HLA-B antigens between mother and child (MW; p=0.002; **Figure 3B**). In addition, the highest HLA-B eplet mismatch loads were found in women with well-controlled SBP (SC; R= -0.389, p=0.0449; **Figure 3C**). Similar negative correlation was found for pulse pressure (difference between SBP and DBP), which is considered a risk factor for cardiovascular disease when > 50 (SC; R= -0.4858, p=0.0102; **Figure 3D**).

**Figure 4:**
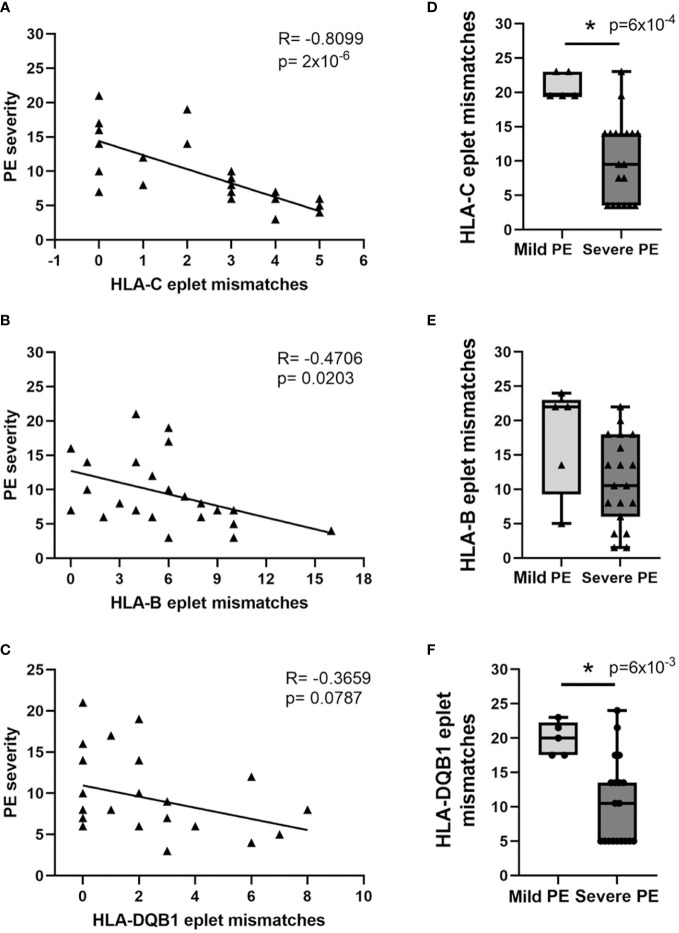
High HLA-C, - B, and -DQB1 eplet incompatibility is associated with milder manifestation of preeclampsia. HLA-C **(A)**, HLA-B **(B)**, and HLA-DQB1 **(C)** eplet incompatibility correlates negatively with PE severity. PE severity was determined according to our scoring system described in the Methods section and reflects the sum of the scores assigned for each parameter on the scoring list. Correlations were calculated with Spearman’s rank correlation, and R and p values are given (n=24). PE stratification into mild (score ≤5, n=5) and severe cases (score >5, n=19) showed significant differences in terms of HLA-C **(D)**, HLA-B **(E)**, and HLA-DQB1 **(F)** eplet mismatch loads between these subgroups. Differences between the subgroups were calculated with the Mann-Whitney U test (n=24). The boxplots depict relative ranks. The medians (symbol within the boxes), quartiles (box), and ranges (whiskers) are shown. *p<0.05 is considered statistically significant.

We also stratified PE patients according to the disease severity with a scoring system described in detail in the *Materials and Methods* section. Interestingly, high mother-child HLA-C, -B, and -DQB1 eplet incompatibility was associated with lower PE severity. The strongest correlation was observed for HLA-C (SC; R= -0.8099, p= 2x10^-6^, **Figure 4A**), then for HLA-B (SC; R= -0.4706, p= 0.0203, **Figure 4B**) and HLA-DQB1 (SC; R= -0.3659, p=0.0787, **Figure 4C**) incompatibilities. Subsequently, based on our scoring system, the patients were divided into mild and severe PE subgroups. A score of 5 was established as a cutoff for mild and severe PE cases. According to this stratification, significantly higher HLA-C (MW; p=6x10^-4^, **Figure 4D**) and HLA-DQB1 (MW; p=6x10^-3^, **Figure 4F**) eplet mismatch loads were hallmarks of mild PE manifestation. A similar trend was observed for HLA-B but did not reach statistical significance (MW; p=0.0883, **Figure 4E**). At least two, four, and one maternal-fetal eplet mismatch was found for HLA-B, -C, and -DQB1 antigens, respectively, in women with milder PE manifestation (**Figure 4**). No differences between mild and severe PE subgroups were found for HLA-A and HLA-DRB1 eplet incompatibilities (data not shown).

### 3.2 Quantity and quality of maternal-fetal HLA eplet mismatches affect preeclampsia severity

**Figure 5:**
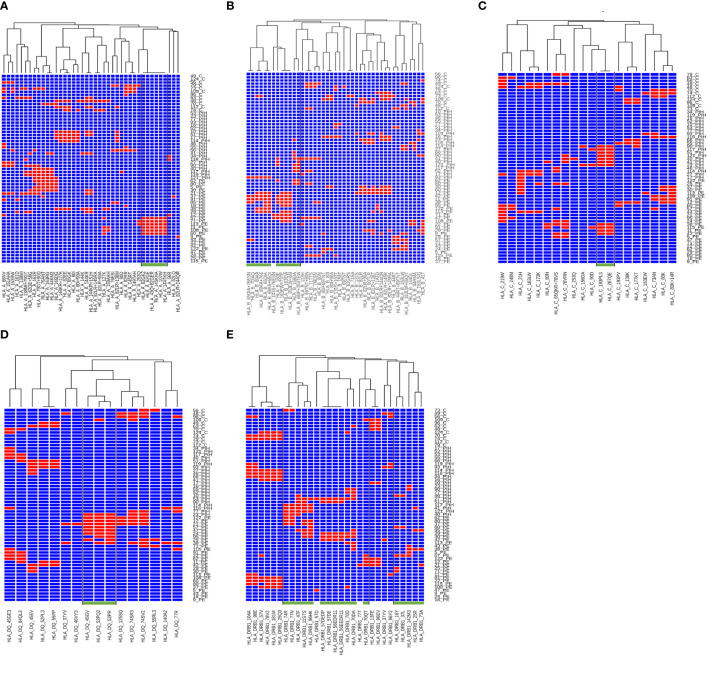
Particular maternal-fetal HLA-A, -B, -C, -DQB1, and -DRB1 eplet mismatches are observed only in pregnancies complicated with GH and PE. The heatmaps depict the presence (red) and absence (blue) of particular HLA-A **(A)**, HLA-B **(B)**, HLA-C **(C)**, HLA-DQB1 **(D)**, and HLA-DRB1 **(E)** eplet mismatches between mother and child in healthy (-C, n=12), gestational hypertension (-PIH, n=22), and preeclampsia (PE, n=27) groups. At the right side of each heat map, the patient ID and group code (-C, -PIH and -PE) are given. At the bottom of each heat map, the eplet names and HLA types are shown. The eplet mismatches that were not present in uncomplicated pregnancies are marked with a green line. Hierarchical clustering according to eplet appearance in studied cases was applied.

As higher eplet mismatch loads between mother and child for HLA-C, -DQB1, and -B antigens were associated with milder manifestation of PE, we decided to check if any particular eplet incompatibility was more frequent in complicated pregnancies and if these eplet mismatches could have prognostic value. For this purpose, we used heat maps to visualize all HLA eplet mismatches identified in each of the studied individuals (**Figure 5**).

Surprisingly, some mother-child eplet mismatches were not observed in physiological pregnancies, but only in those complicated with PE and/or GH. In addition, two, one, and four eplet mismatches in HLA-B, -DQB1, and -DRB1 antigens, respectively, were frequent in GH and PE but detected only in one uncomplicated pregnancy (**Figure 5**). The eplet incompatibilities unique for GH and PE complicated pregnancies are marked with a green line in **Figure 5**. We divided these eplet incompatibilities into five groups that corresponded to the HLA-A, -B, -C, -DRB1, and -DQ antigens where they were identified (**Figure 5**). For HLA-A antigens, the following six eplet mismatches were absent in uncomplicated pregnancies and present as a set in our PE patients: 62GK2, 62GE, 43Q+62GER, 145KHA, 107W, and 144TKH (Group 1 in **Table 5**; **Figure 5A**). In the case of HLA-B, the following 12 eplets were not detected in healthy individuals: 65QIA+76ESN, 70IAQ, 65QIA, 69AA+65QI, 69AA, 69AA+76E, 180E, 163EW+73TE, 163EW, 163EW+66I, 156DA, and 66IF+163TEW (Group a in **Table 5**; **Figure 5B**). The lowest numbers of mother-child eplet mismatches that were present in GH/PE, but not in physiological pregnancies were observed for HLA-C (193PL3 and 267QE, Group 3 in **Table 5**; **Figure 5C**) and HLA-DQB1 (45GV, 52PQ2, and 52PR, Group 4 in **Table 5**; **Figure 5D**). Despite no statistically significant correlations being found between HLA-DRB1 eplet mismatch load and PE severity, 17 mother-child HLA-DRB1 eplet mismatches, namely, 74R, 77N, 47F, 11STS, 96HK, 57DEDP, 57DE, 56EDR11, 56EEDR11, 70D, 70DA, 70QT, 16Y, 37L, 142M3, 25R, and 73A, were identified as characteristic for PIH and PE (Group 5 in **Table 5**; **Figure 5E**). Interestingly, not a single eplet mismatch was identified; instead a defined set of eplet mismatches was detected in GH and PE complicated pregnancies (**Table 5**).

**Table 5:** HLA class I and class II eplet mismatches that were detected only in GH and PE complicated pregnancies and their association with PE severity.

	Eplet/Eplet group	Frequency of eplet incompatibility in the control group (%)	Frequency of eplet incompatibility in the GH group (%)	Frequency of eplet incompatibility in the PE group (%)	§Gestational age of delivery in the presence/absence of a given eplet incompatibility (median and range)	§UPCR [mg/mmol] in the presence/absence of a given eplet incompatibility (median and range)	§PE severity in the presence/absence of a given eplet incompatibility (median and range)
**Group 1** **(HLA-A)**	62GK2 62GE 43Q+62GER 145KHA 107W 144TKH	0/12 (0%)	0/22 (0%)	6/27 (22.2%)	264(233-278)/ 262(190-281)	123.33(22.22-424.92)/ 111.87(13.01-1211.41)	6.5(3-8)/ 9(3-22)
**Group 2** **(HLA-B)**	65QIA+76ESN 70IAO	0/12 (0%)	1/22 (4.5%)	4/27 (14.8%)	267.5(233-278)/262(190-281)	36.67(22.22-101.28)/ 127.72(13.01-1211.41)	3(3-5)/ 9(4-22)*
	65QIA 69AA+65QI	0/12 (0%)	1/22 (4.5%)	7/27 (25.9%)	264(243-278)/257.5(190-281)	101.28(29.61-558.06)/ 119.52(13.01-1211.41)	6(3-19)/ 8.5(3-22)
	65QIA 69AA+65QI 69AA 69AA+76E	0/12 (0%)	1/22 (4.5%)	5/27 (18.5%)	270(243-278)/260(190-281)	101.28(43.73-558.06)/ 119.52(13.01-1211.41)	5.5(3-12)/ 8.5(3-22)
	180E	0/12 (0%)	3/22 (13.6%)	5/27 (18.5%)	270.5(264-278)/253(190-281)*	72.5(21.38-307.43)/ 127.72(13.01-1211.41)	5(3-7)/ 9(3-22)*
	163EW+73TE 163EW 163EW+66I	0/12 (0%)	0/22 (0%)	9/27 (33.3%)	270.5(243-278)/251(190-281)* P=0.04	127.17(29.61-424.92)/ 88.35(13.01-1211.41)	5(3-7)/ 9(3-22)
	156DA 66IF+163TEW	0/12 (0%)	4/22 (18.2%)	2/27 (7.4%)	233(198-268)/262(190-281)	120.01(21.38-218.64)/ 111.87(13.01-1211.41)	22
**Group 3 (HLA-C)**	193PL3 267QE	0/12 (0%)	7/22 (31.8%)	4/27 (14.8%)	270(264-278)/ 253(190-281)	72.5(29.61-111.87)/ 127.7(13.01-1211.41)	5(3-6)/ 9(3-22)*
**Group 4** **(HLA-DQB1)**	45GV 52PQ2 52PR	0/12 (0%)	1/22 (4.5%)	6/27 (22.2%)	258(233-276)/ 262(190-281)	69.83(15.95-558.06)/127.72 (13.01-1211.41)	7.5(3-12)/ 8.5(3-22)
	52PQ2 52PR	0/12 (0%)	1/22 (4.5%)	8/27 (29.6%)	262.5(233-276)/ 262(190-281)	69.83(15.95-558.06)/ 145.39(13.01-1211.41)	7.5(3-19)/ 8.5(3-22)
**Group 5** **(HLA-DRB1)**	74R 77N	1/12 (8.3%)	4/22 (18.2%)	3/27 (11.1%)	198(190-268)/ 262.5(221-281)	48.78(21.38-218.64)/ 119.52(13.01-1211.41)	14.5(7-22)/ 8(3-19)
	47F	0/12 (0%)	7/22 (31.8%)	1/27 (3.7%)	262	127.72	19
	11STS 96HK	0/12 (0%)	4/22 (18.2%)	5/27 (18.5%)	262(190-279)/262.5(221-281)	218.64(48.78-249.15)/88.05(13.01-1211.41)	18(7-22)/ 7(3-16)
	57DEDP 57DE 56EDR11 56EEDR11	0/12 (0%)	2/22 (9.1%)	3/27 (11.1%)	271(265-279)/ 257.5(190-281)	237.06(43.73- 249.15)/ 106.575(13.01-1211.41)	9(3-18)/ 8(3-22)
	70D	1/12 (8.3%)	3/22 (13.6%)	4/27 (14.8%)	263.5(243-279)/262(190-281)	243.10(145.39-424.92)/ 74.83(13.01-1211.41)	8.5(7-18)/ 7.5(3-22)
	70DA	1/12 (8.3%)	4/22 (18.2%)	6/27 (22.2%)	263(224-279)/262(190-281)	136.55(29.61-249.15)/101.28(13.01-1211.41)	8(5-19)/ 8(3-22)
	70QT	1/12 (8.3%)	0/22 (0%)	4/27 (14.8%)	249.5(233-270)/ 263(190-281)	69.83(22.22-558.06)/127.17(13.01-1211.41)	6(3-12)/ 8.5(3-22)
	16Y	0/12 (0%)	1/22 (4.5%)	4/27 (14.8%)	268(233-281)/ 262(190-279)	45.89(22.22-307.43)/ 127.17(13.01-1211.41)	6(3-6)/ 9(3-22)*
	37L	0/12 (0%)	1/22 (4.5%)	2/27 (7.4%)	249.5(233-266)/ 262(190-281)	26.15(22.22-30.09)/ 127.17(13.01-1211.41)	4.5(3-6)/ 8.5(3-22)
	142M3	0/12 (0%)	2/22 (9.1%)	4/27 (14.8%)	241(224-264)/263(190-281)	42.71(22.22-610.21)/ 127.17(13.01-1211.41)	6(3-14)/ 5(3-22)
	25R	0/12 (0%)	1/22 (4.5%)	2/27 (7.4%)	263(262-264)/ 262(190-281)	78.66(29.61-127.72)/111.87(13.01-1211.41)	12(5-19)/ 8(3-22)
	73A	0/12 (0%)	0/22 (0%)	3/27 (11.1%)	262(253-264)/ 262.5(190-281)	64.84(29.61-127.72)/ 119.52(13.01-1211.41)	8(5-19)/ 8(3-22)

The table depicts the frequency of the presence of a given maternal-fetal HLA eplet mismatch in the control groups and pregnancies with gestational hypertension (GH) and preeclampsia (PE). The eplet mismatches that were not present in uncomplicated pregnancies were divided into five groups according to the type of HLA where the eplets were detected. Median and range of gestational age at delivery, urine protein creatinine ratio (UPCR), and PE severity in pregnancies with PE in the presence/absence of a given eplet mismatch group are also shown. §analysis for women with PE; *p<0.05, MW test. #Statistically significant differences in PE severity in case of presence and absence of a given eplet group are depicted with red font and marked with "*"; *p<0.05. Data for the control, GH and PE groups are shaded with blue, yellow and red colours, respectively.

Unexpectedly, the eplet mismatches that were characteristic only for complicated pregnancies were associated with milder PE. Mismatches in 65QIA+76ESN and 70IAO or in 180E HLA-B eplet in the PE group were associated with lower severity of PE (score 3 vs 9, MW; p= 0.001 and score 5 vs 9, MW; p=0.04; respectively, **Table 5**). In addition, incompatibility in 180E HLA-B eplet, as well as simultaneous mismatch in 163EW+73TE, 163EW, and 163EW+66I HLA-B eplets, were a hallmark of lower risk of premature delivery (median gestational age at birth 270.5 vs 253, MW; p= 0.006 and 270.5 vs 251, MW; p= 0.04, respectively, **Table 5**). Lower severity of PE was also associated with the presence of mismatches in 193PL3 and 267QE HLA-C eplets (score 5 vs 9, MW; p= 0.01, **Table 5**), as well as in 16Y eplet present in HLA-DRB1 antigens (score 6 vs 9, MW; p= 0.03, **Table 5**). In addition, no FGR was observed in pregnancies with 193PL3 and 267QE HLA-C eplet mismatches.

### 3.3 Presence of anti-HLA antibodies is not a hallmark of GH and PE

Anti-HLA-A antibodies (Abs) were detected in 2/12 (16.66%) women from the control group, 1/22 (4.54%) woman in the GH group, and 2/27 (7.4%) women in the PE group. The frequencies of anti-HLA-B Abs in the control, GH, and PE groups were as follows: 2/12 (16.66%), 1/22 (4.54%), and 4/27 (14.81%), respectively. No anti-HLA-C Abs were detected for any of the studied individuals. Anti-HLA-DRB1 Abs were found in 1/12 (8.33%), 2/22 (9.09%), and 2/27(7.4%) mothers in the control, GH, and PE cohorts, respectively. The presence of anti-MICA Abs was analyzed in some individuals and its frequency for the control, GH, and PE groups was: 1/7 (14.28%), 2/14 (14.28%), and 4/20 (20%), respectively (**Table 6**). No statistically significant differences in terms of anti-HLA and anti-MICA Abs were found between the groups.

**Table 6:** Anti-fetal HLA class I, class II, and anti-MICA antibodies detected in maternal sera in the control, GH, and PE groups.

Group	Patient no.	Anti-HLA-A Abs	Anti-HLA-B Abs	Anti-HLA-C Abs	Anti- HLA-DRB1 Abs	Anti- HLA-DQB1 Abs	Anti-MICA Abs
**Control group**	1	+	+	–	-	-	-
	2	–	–	–	-	-	-
	3	–	–	–	-	-	-
	4	–	–	–	-	-	+
	5	–	–	–	-	-	-
	6	–	–	–	-	-	-
	7	–	–	–	-	-	-
	8	–	–	–	-	-	N/A
	9	–	–	–	-	-	N/A
	10	+	+	–	+	-	N/A
	11	–	–	–	-	-	N/A
	12	–	–	–	-	-	N/A
**No. of women with anti-HLA and anti-MICA Abs**		**2**	**2**	**0**	**1**	**0**	**1**
**GH**	13	–	–	–	-	-	N/A
	14	–	–	–	-	-	+
	15	–	–	–	-	-	-
	16	–	–	–	-	-	-
	17	–	–	–	-	-	+
	18	–	+	–	+	+	-
	19	–	–	–	-	-	-
	20	+	–	–	+	-	-
	21	–	–	–	-	-	-
	22	–	–	–	-	-	-
	23	–	–	–	-	-	-
	24	–	–	–	-	-	-
	25	–	–	–	-	-	-
	26	–	–	–	-	-	-
	27	–	–	–	-	-	-
	28	–	–	–	-	-	N/A
	29	–	–	–	-	-	N/A
	30	–	–	–	-	-	N/A
	31	–	–	–	-	-	N/A
	32	–	–	–	-	-	N/A
	33	–	–	–	-	-	N/A
	34	–	–	–	-	-	N/A
	35	–	–	–	-	-	N/A
**No. of women with anti-HLA and anti-MICA Abs**		**1**	**1**	**0**	**2**	**1**	**2**
**PE**	36	–	–	–	-	-	-
	37	–	+	–	-	-	-
	38	–	+	–	+	-	-
	39	–	–	–	-	-	+
	40	–	–	–	-	-	-
	41	–	–	–	-	-	+
	42	–	+	–	-	-	-
	43	–	–	–	-	-	-
	44	–	–	–	-	-	+
	45	–	–	–	-	-	-
	46	+	–	–	-	-	-
	47	–	–	–	-	-	-
	48	–	+	–	+	-	+
	49	–	–	–	-	-	-
	50	–	–	–	-	-	-
	51	–	–	–	-	-	-
	52	–	–	–	-	-	-
	53	–	–	–	-	-	-
	54	–	–	–	-	-	-
	55	–	–	–	-	-	-
	56	–	–	–	-	-	N/A
	57	–	–	–	-	-	N/A
	58	–	–	–	-	-	N/A
	59	+	–	–	-	-	N/A
	60	–	–	–	-	-	N/A
	61	–	–	–	-	-	N/A
**No. of women with anti-HLA and anti-MICA Abs**		**2**	**4**	**0**	**2**	**0**	**4**

The table depicts if antibodies (Abs) against HLA class I (white), HLA class II (blue), and MICA (non-classical HLA) antigens (grey) were detected in the maternal sera. Numbers of the women positive for a given anti-HLA Ab are also shown for each group. Anti-MICA Abs were analyzed only for selected individuals. Non-examined samples are marked as non-applicable (N/A). + and – correspond to the presence and absence of anti-HLA Abs, respectively.

## 4 Discussion

In the present study, we found that higher HLA-B, -C, and -DQB1 eplet compatibility between mother and child was associated with higher PE severity. We also identified several eplet mismatches that were unique to GH and PE and were not detected in uncomplicated pregnancies. Unexpectedly, these mismatches were associated with milder manifestation of PE, when PE occurred. Induction of anti-fetal HLA antibodies in the mother was not associated with the defined eplet mismatches or PE onset and severity.

Approximately 50% of idiopathic cases of recurrent miscarriages have immune etiology (35, 36). It is also widely accepted that proper maternal tolerance towards developing embryos is crucial for implantation and successful pregnancy. Nevertheless, these mechanisms are still poorly understood (35) and were ignored as possible etiologic factors for PE till the early 90s. This probably resulted from the high heterogeneity of the studies’ designs and focus on maternal-paternal rather than maternal-fetal HLA compatibility (37).

In 2010, Biggar RJ et al. hypothesized that high maternal-fetal HLA incompatibility might be responsible for PE onset. Nevertheless, intermediate-level typing of HLA- A, -B, and -DR antigens from physiologic and PE complicated pregnancies did not confirm that hypothesis (32). However, only high-resolution HLA typing (such as NGS performed in our study) enables detailed analysis of HLA mismatches at the eplet level. While eplets are directly recognized by immune cells and antibodies, they determine the immunogenicity of a particular HLA incompatibility (25, 34).

Till now only one group analyzed eplets in pregnancy. Honger et al. reported several highly immunogenic HLA-A eplets in uncomplicated pregnancies and those that did not induce anti-HLA antibodies (34). Interestingly, 4 (145KHA, 144TKH, 62GE, and 107W) per 10 (62GK, 145KHA, 144TKH, 62GE, 107W, 80I, 82LR, 41T, 127K, and 45KE) of these immunogenic eplets overlap with HLA-A eplet mismatches unique for PE in our study and were present in 22.2% cases. However, we did not find any correlation between these eplet incompatibilities and anti-HLA antibody induction. We also did not observe any protective effect of eplets designated as non-reactive by Honger et al. (62RR, 76SN, 80TLR, 156DA, and 163RW) (34).

Our study is the first where maternal-fetal eplet mismatches were analyzed for both HLA class I and class II antigens. Unexpectedly, we observed that low HLA-A, -B, -C, and -DQB1 incompatibility between mother and child is associated with higher PE severity. The most significant correlations were found for HLA-C eplet mismatch load. It is known that HLA-C are the only classical HLA molecules expressed on EVTs that are of fetal origin (38, 39). Thus, HLA-C is the key molecule that can elicit allogeneic immune responses by maternal T and NK cells and is also the main reason why maternal-fetal immune tolerance needs to be established (39).

Recently, Creaenmehr et al. suggested that uncomplicated pregnancy outcome requires semi-compatibility between HLA-DR antigens (40). These results are in line with previous observations for heart and kidney transplantation. The allograft survival was improved if transplantation was preceded by blood transfusion from a donor with one HLA-DR antigen mismatch as compared with individuals who were not subjected to blood transfusion (41). These data indicate that immune tolerance to foreign antigens is a complex process developing with time when proper stimulation is delivered to the immune system.

In the current study, we showed for the first time that high HLA-B, -C, and -DQB1 incompatibilities between mother and child have a protective effect in PE. The effect was the most pronounced for HLA-C. A complete lack of HLA-C mismatches was observed in 25% of severe PE cases. On the contrary, mild PE was characterized by ≥4 HLA-C eplet mismatches. In addition, we determined five sets of maternal-fetal eplet incompatibilities common for GH and PE but that were not observed in physiological pregnancies. Thus, the profile of maternal-fetal HLA eplet mismatches and their number have prognostic values in GH and PE. Further research on eplet incompatibility in the context of immune cell activation will result in a deeper understanding of tolerance development in semi-allogenic fetuses. This knowledge is indispensable for the effective treatment of various pregnancy complications, including immune-mediated miscarriages or antiphospholipid syndrome. We are also convinced that induction of pregnancy-like immune tolerance in allograft recipients may lead to immunosuppression-free graft survival. It is known that interactions between dNK cells and fetal HLA-C and HLA-G molecules expressed on EVT cells contribute to trophoblast invasiveness, vascular remodeling, and induction of fetal tolerance (38, 39). Nevertheless, our study is the first that indicates the importance of maternal-fetal HLA eplet incompatibility in this process and suggests its protective function in PE.

We are convinced that analysis of HLA eplet mismatches is a new direction for studies on the immunology of pregnancy. With this approach, we will be able to explore the effect of defined HLA eplet incompatibility on pregnancy outcome and severity of potential complications. Undoubtedly, both quantity and quality of HLA eplet mismatches shape maternal-fetal tolerance and future studies will complement this knowledge.

The main strength of the present study is the originality of the research approach. Previously low or intermediate-resolution HLA typing was used in studies on pregnancy complications and no data on eplet compatibility in GH and PE were reported. Our results have prognostic and predictive value. Nevertheless, exploitation of the method in clinical practice requires fetal blood or amniocyte collection before GH onset. Therefore, the accessibility of fetal DNA is the main obstacle to the use of this method in daily practice.

Despite this limitation, we suggest that the present research paper will initiate a new trend in studies on the immunology of pregnancy, contributing to understanding how the quantity and quality of HLA mismatches shape maternal-fetal tolerance.

High HLA-C, -B, and DQB1 eplet compatibility between mother and fetus is associated with severe PE manifestation. In addition, high HLA-B and HLA-DQB1 compatibility is associated with earlier PE onset and, as a consequence, preterm birth. Both quantity and quality of HLA eplet mismatches affect the severity of PE. Mismatches in HLA-B eplets: 65QIA+76ESN, 70IAO, and 180E, HLA-C eplets: 193PL3 and 267QE, and HLA-DRB1 eplet: 16Y were associated with mild outcomes of PE when PE occurred. HLA incompatibility between mother and offspring is crucial for the induction of tolerance to the semi-allogenic fetus and is required for the physiological outcome of pregnancy, supporting the theory that high diversity is evolutionarily preferred.

## Data availability statement

The datasets for this article are not publicly available due to concerns regarding patient anonymity. Requests to access the datasets should be directed to the corresponding author.

## Ethics statement

The study was approved by Independent Bioethics Commission for Research of the Medical University of Gdańsk (agreement no. NKBBN/454/2014). It was conducted in accordance with the European and local legislation and institutional requirements. Written informed consent for participation in this study was provided by the adults and by the legal guardians/next of kin in case of the children.

## Author contributions

KS: Conceptualization, Funding acquisition, Investigation, Project administration, Resources, Writing – original draft. MK: Investigation, Methodology, Visualization, Software, Writing – original draft. KPi: Investigation. EC: Formal analysis. DZ: Resources, Investigation. JJ-B: Resources, Investigation. PA: Resources, Investigation. RŚ-S: Resources, Investigation. AA-C: Resources, Investigation. KL: Resources, Investigation. MZ: Methodology. KP: Resources, Investigation. HZ: Methodology, Software. BT: Methodology, Investigation, Data curation. PT: Conceptualization, Methodology. NM-T: Conceptualization, Data curation, Formal analysis, Funding acquisition, Investigation, Methodology, Project administration, Resources, Supervision, Validation, Visualization, Writing – original draft, Writing – review & editing.
